# *Fucus vesiculosus* and *Ascophyllum nodosum* Ameliorate Liver Function by Reducing Diet-Induced Steatosis in Rats

**DOI:** 10.3390/md18010062

**Published:** 2020-01-17

**Authors:** Daniela Gabbia, Miriam Saponaro, Samantha Sarcognato, Maria Guido, Nicola Ferri, Maria Carrara, Sara De Martin

**Affiliations:** 1 Department of Pharmaceutical and Pharmacological Sciences, University of Padova, 35100 Padova, Italy; daniela.gabbia@unipd.it (D.G.); nicola.ferri@unipd.it (N.F.); maria.carrara@unipd.it (M.C.); 2 Department of Medicine, University of Padova, 35100 Padova, Italy; miriam.saponaro@outlook.it; 3 Venetian Institute of Molecular Medicine—VIMM, 35100 Padova, Italy; 4 Department of Medicine, General Pathology and Cytophatology Unit, University of Padova, 35100 Padova, Italy; samantha.sarcognato@gmail.com (S.S.); mguido@unipd.it (M.G.); 5 Department of Pathology, Azienda ULSS2 Marca Trevigiana, 31100 Treviso, Italy

**Keywords:** *Fucus vesiculosus*, *Ascophyllum nodosum*, brown seaweeds, NAFLD, insulin resistance, blood glucose

## Abstract

The Asian coastal communities have used the brown seaweeds *Fucus vesiculosus* and *Ascophyllum nodosum* since ancient times. Recently, some in vitro and in vivo studies have demonstrated their abilities in reducing risk factors for metabolic syndrome. Here, we analyzed the protective effect of a phytocomplex extracted from these seaweeds on the deposition of fat in the liver after the administration of a high-fat diet (HFD) to rats for five weeks. The administration of *F. vesiculosus* and *A. nodosum* led to significant reductions in microvescicular steatosis and plasma biochemical and lipid parameters, such as alanine aminotransferase (ALT), aspartate aminotransferase (AST), alkaline phosphatase (ALP), total and conjugated bilirubin, and triglycerides. Furthermore, the postprandial glycemic peak was delayed and significantly reduced (*p* < 0.01) by the algal extract administration. In conclusion, this extract is effective in reducing microvescicular steatosis and improving glycemic control, thereby lowering the risk of nonalcoholic fatty liver disease, obesity, and diabetes, diseases related to the consumption of fat and sugar-enriched diets.

## 1. Introduction

The brown seaweeds *Fucus vesiculosus* and *Ascophyllum nodosum* are easily available food sources that have been used since ancient times by the coastal communities of Britain, Asia, and other countries [1]. A number of studies have recently confirmed that the consumption of seaweeds ameliorates the conditions of patients affected by metabolic diseases, e.g., type 2 diabetes (T2D) and obesity [2,3], by virtue of their numerous bioactive compounds, such as phlorotannins and fucoidans [4,5,6,7,8].

The modern western lifestyle is characterized by a drastic increase in the consumption of high-palatable hypercaloric diets, which lead to obesity, T2D, and metabolic syndrome [9]. This has been associated with metabolic disorders, e.g., nonalcoholic fatty liver disease (NAFLD), but also with pathologic conditions characterized by a low-grade inflammatory state, which could lead to severe immune and cognitive dysfunctions [10,11,12].

In this context, a behavioral approach aimed at lifestyle modifications could address this complex interplay of alterations. In particular, nutrition plays a pivotal role in the treatment of these metabolic alterations, considering that it is well-known that an appropriate intake of energy can improve all these conditions. It has been demonstrated that *Fucus vesiculosus* and *Ascophyllum nodosum* are rich in natural compounds able to slow down cholesterol absorption by increasing intestinal viscosity and delaying and decreasing sugar absorption through the inhibition of both α-amylase and α-glucosidase [5,13,14,15]. In particular, it has already been reported that this inhibitory effect is due to the high content of different bioactive compounds in the algal extract, and the fingerprint analysis we performed in our previous study indicated the presence of three main types of constituents, i.e., polysaccharides, polyphenolics, and fatty acids [5].

On the basis of these considerations, the aim of this study was to ascertain whether a chronic administration of a hot water extract obtained from *Fucus vesiculosus* and *Ascophyllum nodosum* may be useful in preventing high-fat diet-induced fatty livers and their associated dysregulation of glycemic control.

## 2. Results

### 2.1. Body and Liver Weights

**Figure 1:**
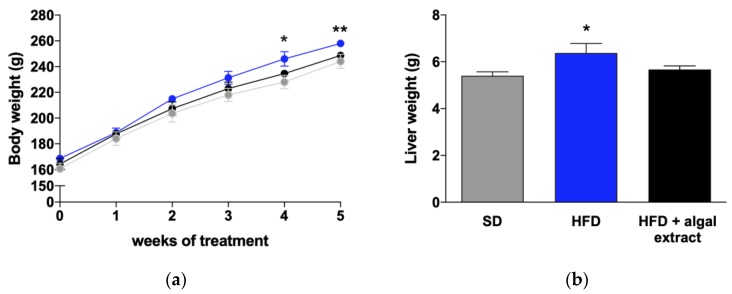
Body weights (g) of rats during the 5 weeks of treatment (**a**) and liver weights (**b**) at sacrifice. Grey line = rats fed with standard diet (SD), blue line = rats fed with high-fat diet (HFD), and black line = rats fed with HFD + algal extract. Data are reported as mean ± S.E.M. * = *p* < 0.05 and ** = *p* < 0.01 vs. rats fed with SD.

As shown in Figure 1a, the body weights of rats fed with high-fat diet (HFD) were higher than that of animals fed with standard diet (SD), starting from the second week of treatment, and this difference reached statistical significance after four weeks. The weights of HFD rats treated with the algal extract were virtually identical to that of control rats. Similarly, at the moment of sacrifice, the liver weights of rats fed with HFD were significantly higher than that of controls, while those of rats treated with the algal extract did not differ from controls (Figure 1b).

### 2.2. Liver Histology and Plasma Biochemistry

**Figure 2:**
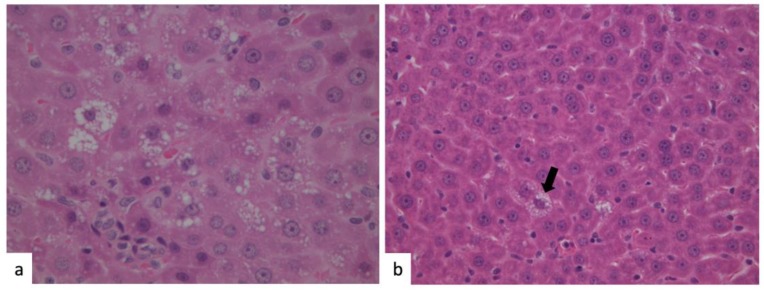
Liver histology. Hematoxylin–eosin (H&E) staining of liver tissues obtained from rats fed with HFD (**a**) and HFD with algal phytocomplex (**b**). A diffuse microvescicular steatosis could be observed in rats fed with HFD (**a**), whereas very rare cells with microvescicular steatosis could be evidenced in the rats treated with the algal extract ((**b**), black arrow).

In order to evaluate liver status, we performed a histological analysis of liver tissues by hematoxylin–eosin staining of paraffine-embedded liver slices and measuring biochemical markers of liver function. During histology, moderate steatosis, mainly microvescicular, was observed in HFD rats (Figure 2a), while only isolated steatotic hepatocytes were found in the animals treated with the algal extract (Figure 2b, arrow).

**Figure 3:**
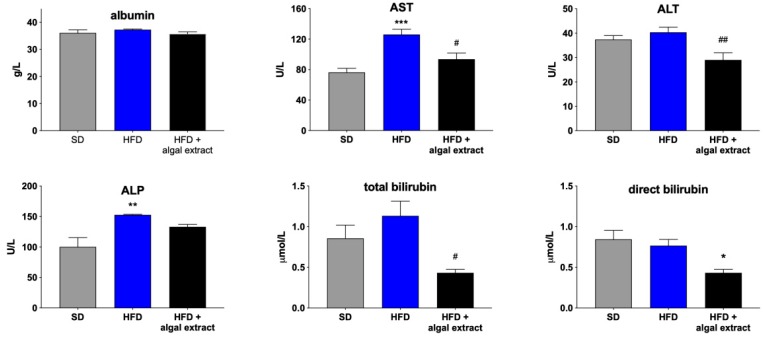
Biochemical markers of liver function in rats fed with standard (SD), high-fat diet (HFD), and high-fat diet treated with the algal extract. ALT: alanine aminotransferase; AST: aspartate aminotransferase; ALP: alkaline phosphatase. * = *p* < 0.05, ** = *p* < 0.01 and *** = *p* < 0.001 vs. rats fed with SD; ^#^ = *p* < 0.05 and ^##^ = *p* < 0.01 vs. rats fed with HFD.

Coherently, lower levels of serum biochemical parameters were observed in the treated rats (Figure 3). In particular, alanine aminotransferase (ALT), aspartate aminotransferase (AST), and total and conjugated bilirubin were significantly decreased by the treatment with respect to HFD rats, suggesting its beneficial role in liver function.

**Figure 4:**
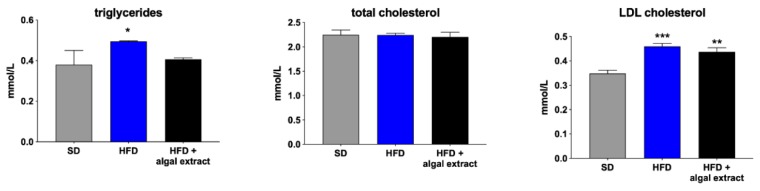
Plasma lipid profile of rats fed with standard (SD), high-fat diet (HFD), and high-fat diet treated with the algal extract. * = *p* < 0.05, ** = *p* < 0.01, and *** = *p* < 0.001 vs. rats fed with SD.

As expected, plasma triglycerides and low-density lipoproteins (LDL) cholesterol were significantly higher in HFD rats when compared to animals fed with standard diet (Figure 4). The treatment with the algal extract reduced only plasma triglycerides, whereas it had no effects on total and LDL cholesterol.

### 2.3. Postprandial Blood Glucose Levels

**Figure 5:**
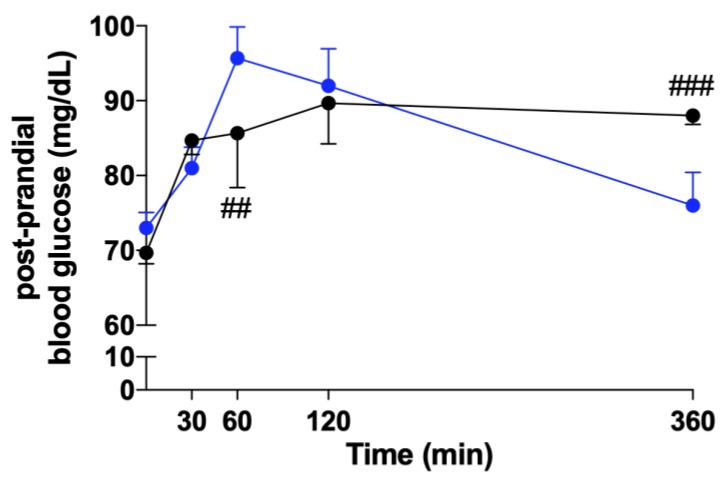
Postprandial blood glucose levels after starch ingestion. After an overnight fast, rats were fed by oral gavage with a 50%–50% starch and sunflower oil solution, and blood glucose levels were measured at 30, 60, 120, and 360 min. Blue line = mice fed with HFD and black line = mice fed with HFD + algal extract. Data are reported as mean ± S.E.M. ^##^ = *p* < 0.01 and ^###^ = *p* < 0.001 vs. rats fed with HFD.

We measured postprandial blood glucose levels in the two groups of HFD rats after a starch-simulated high-carbohydrate meal. As shown in Figure 5, the treatment with the algal extract affects level and timing of postprandial blood glucose peak, since it was delayed (120 vs. 60 min) and reduced (89.67 ± 13.27 vs. 95.67 ± 10.30 mg/dL) by the treatment. Notably, blood glucose of treated animals was significantly lower (*p* < 0.01) than that of the untreated group 60 min after the meal, whereas it was significantly higher after 360 min (*p* < 0.001), confirming that the algal components act by delaying carbohydrate digestion and sugar absorption.

## 3. Discussion

In this study, we investigated the effects of the five-week administration of the phytocomplex obtained from two brown algae, *A. nodosum* and *F. vesiculosus*, on glycemic control and liver function of HFD-treated Wistar rats. Several studies have confirmed the multiple beneficial effects of seaweed products, since their bioactive components have antidiabetic, antiobesity, and antioxidant properties [4,13,16,17,18,19,20]. A recent study reported that ascophyllan from *A. nodosum* is able to induce glucagon-like peptide-1 (GLP-1) secretion from human intestinal cells, suggesting that it may represent a useful agent for controlling blood glucose in humans [21,22]. In Western countries, widespread consumption of hypercaloric diets has been registered in the last decades, leading to increased incidences of metabolic diseases, such as obesity, T2D, and metabolic syndrome [9]. Since appropriate intake of energy can improve these clinical conditions, nutritional adjustments, such as reductions of glucose intake, might play a pivotal role in the clinical management of these metabolic alterations. The inhibition of the two intestinal enzymes α-amylase and α-glucosidase, both involved in carbohydrate digestion, can significantly decrease and delay glucose absorption after a composite carbohydrate meal. Accordingly, synthetic α-glucosidase inhibitors, such as acarbose, are currently used in the treatment of T2D. Some studies have already demonstrated that specific components of *F. vesiculosus* and *A. nodosum* (phlorotannins and fucoidans) inhibit both α-amylase and α-glucosidase, thereby delaying/decreasing sugar absorption [5,13,14,15,21].

The findings we obtained about plasma bilirubin levels deserve further discussion. Indeed, it is well-known that a controversial relationship exists between plasma bilirubin and NAFLD, since, although a mild increase of serum bilirubin levels had been associated with a reduction of liver steatosis risk [23], a more recent study [24] provided convincing evidences against this hypothesis. Our data indicate that the algal extract decreases bilirubin plasma levels, as well as ALT and AST, thereby confirming its beneficial effects on liver function, which are also demonstrated by histology. On the contrary, dietary supplements based on *Silybum marianum* (milk thistle), a plant with a well-recognized antioxidant [25] and hepatoprotective actions (see, e.g., [26] and references therein) have been demonstrated to improve liver function by increasing bilirubin levels and suppressing UGT1A1, the enzyme responsible for bilirubin conjugation, and decreasing body weight [27]. Furthermore, bilirubin was recently found to act through the nuclear receptor PPARα [28,29] to reduce body weight. However, the results obtained in this study are probably the consequence of a different mechanism of action, since, as stated before, the effects of the extract obtained from the brown algae *A. nodosum* and *F. vesiculosus* have been linked to the inhibition of the two digestive enzymes α-amylase and α-glucosidase, which leads to a decrease of glucose absorption. Further pharmacokinetic and mechanistic studies are needed to ascertain whether any algal component is absorbed by the intestines and reaches the liver, acting there by means of different mechanisms.

Taken together, the results of our study (reductions in body weight, free triglycerides, and blood glucose peak of treated animals) confirm previous observations, suggesting an effect of this treatment on insulin resistance, although it has to be noted that the rats involved in this study still had normal glycemic control (Figure 5), probably because of the limited period of HFD feeding. Moreover, our findings notably link these effects to liver function, since we demonstrated for the first time that the administration of a commercial phytocomplex obtained from these two algae is able to reduce liver steatosis and the plasma concentration of several liver function markers, whose increases are associated with hepatic impairment. These findings are extremely relevant, since the prevalence of NAFLD and its complications in nonalcoholic steatohepatitis (NASH) are increasing worldwide, and no therapeutic options are currently available [30].

## 4. Materials and Methods

### 4.1. Algal Extract

The phytocomplex used in this study was extracted from the brown seaweeds *A. nodosum* and *F. vesiculosus* and is commercially available under the name InSea2^®^ (Rimouski, QC, Canada). This extract, titrated in polyphenols at 20%, was prepared by hot water extraction, ultrafiltration processes, and then spray-dried to obtain a brown soluble powder. Its chemical characterization has already been described in detail [5].

### 4.2. Study Design

All the experimental procedures involving animals were in compliance with national and European guidelines for the handling and use of experimental animals (authorization No. 799/2016; 30 August 2016). Ten male Wistar rats, aged 5 weeks (body weight 150–170 g), were obtained from Charles River Italia (Calco, Lecco, Italy). They were kept under controlled environmental conditions and fed with standard diet for 2 weeks before the beginning of the treatment [31]. Then, the animals were randomized into two experimental groups, both fed with high-fat diet (HFD = kcal from 23.5% protein, 18.4% carbohydrate, and 60.3% fat; Altromin; Lage, Germany). During the 5-week administration of HFD, one group of rats was treated daily with an oral administration by intragastric gavage of the algal extract (7.5 mg/kg·bw).

### 4.3. Postprandial Blood Glucose Levels, Biochemical and Histological Analysis

After 5 weeks of treatment, rats were fed with a 50%–50% starch and sunflower oil solution, as previously described [5]. Blood glucose levels were measured 30, 60, 120, and 360 min after the simulated meal by snipping the tails and using a glucose meter (BG Star, MDSS GmbH; Hannover, Germany). After 360 min, rats were sacrificed by excesses of gaseous anesthesia with isoflurane, and blood was collected by intracardiac puncture. In order to evaluate the presence of HFD-induced steatosis, rat livers were collected, weighed, and a histological analysis was performed by means of the standard hematoxylin–eosin staining technique [32] and evaluated by two pathologists (S.S. and M.G.) who were blinded to the treatment of the rats [33]. Liver function was assessed by measuring biochemical markers, i.e., serum concentrations of albumin, alanine aminotransferase (ALT), aspartate aminotransferase (AST), alkaline phosphatase (ALP), and total and conjugated bilirubin [34]. The plasma lipid profile was assessed by measuring triglycerides, total cholesterol, and LDL cholesterol [35]. These plasma biochemical parameters were also measured in 5 rats fed with standard diet. All plasma biochemical parameters were assessed by standard laboratory methods.

### 4.4. Statistical Analysis

All data are expressed as mean ± S.E.M. and analyzed with GraphPad Prism (GraphPad Software Inc.; San Diego, CA, USA) ver. 8.0. Statistical analysis was performed by means of a Student’s *t*-test or one-way ANOVA, followed by Tukey’s *post-hoc* test when appropriate. *p* < 0.05 was considered statistically significant [36,37].

## 5. Conclusions

In conclusion, the algal extract obtained from the brown algae *F. vesiculosus* and *A. nodosum* is effective in reducing body weight and postprandial glucose levels, thereby representing a useful tool for lowering the risk of metabolic diseases related to the consumption of high amounts of dietary fat. Furthermore, since the consumption of this extract has been for the first time associated with a reduction of hepatic fat, further studies are needed to explore the use of brown seaweed extracts in the management of NAFLD and NASH, hepatic diseases often associated with unhealthy eating habitudes.

## References

[1] Rupérez P., Ahrazem O., Leal J.A. (2002). Potential antioxidant capacity of sulfated polysaccharides from the edible marine brown seaweed *Fucus vesiculosus*. J. Agric. Food Chem..

[2] Landin K., Holm G., Tengborn L., Smith U. (1992). Guar gum improves insulin sensitivity, blood lipids, blood pressure, and fibrinolysis in healthy men. Am. J. Clin. Nutr..

[3] Ou S., Kwok K., Li Y., Fu L. (2001). In vitro study of possible role of dietary fiber in lowering postprandial serum glucose. J. Agric. Food Chem..

[4] Apostolidis E., Lee C.M. (2010). In vitro potential of *Ascophyllum nodosum* phenolic antioxidant-mediated α-glucosidase and α-amylase inhibition. J. Food Sci..

[5] Gabbia D., Dall’Acqua S., Di Gangi I.M., Bogialli S., Caputi V., Albertoni L., Marsilio I., Paccagnella N., Carrara M., Giron M.C. (2017). The phytocomplex from *Fucus vesiculosus* and *Ascophyllum nodosum* controls postprandial plasma glucose levels: An in vitro and in vivo study in a mouse model of NASH. Mar. Drugs.

[6] Gupta S., Abu-Ghannam N. (2011). Bioactive potential and possible health effects of edible brown seaweeds. Trends Food Sci. Technol..

[7] Nwosu F., Morris J., Lund V.A., Stewart D., Ross H.A., McDougall G.J. (2011). Anti-proliferative and potential anti-diabetic effects of phenolic-rich extracts from edible marine algae. Food Chem..

[8] Yoshie Y., Wang W., Petillo D., Suzuki T. (2000). Distribution of catechins in Japanese seaweeds. Fish. Sci..

[9] Manchanda S., Kaur G. (2017). *Withania somnifera* leaf alleviates cognitive dysfunction by enhancing hippocampal plasticity in high fat diet induced obesity model. BMC Complement. Altern. Med..

[10] Castanon N., Lasselin J., Capuron L. (2014). Neuropsychiatric comorbidity in obesity: Role of inflammatory processes. Front. Endocrinol..

[11] Craft S. (2005). Insulin resistance syndrome and Alzheimer’s disease: Age- and obesity-related effects on memory, amyloid, and inflammation. Neurobiol. Aging.

[12] Pistell P.J., Morrison C.D., Gupta S., Knight A.G., Keller J.N., Ingram D.K., Bruce-Keller A.J. (2010). Cognitive impairment following high fat diet consumption is associated with brain inflammation. J. Neuroimmunol..

[13] Derosa G., Cicero A.F.G., D’Angelo A., Maffioli P. (2019). *Ascophyllum nodosum* and *Fucus vesiculosus* on glycemic status and on endothelial damage markers in dysglicemic patients. Phytother. Res..

[14] Paradis M.-E., Couture P., Lamarche B. (2011). A randomised crossover placebo-controlled trial investigating the effect of brown seaweed (*Ascophyllum nodosum* and *Fucus vesiculosus*) on postchallenge plasma glucose and insulin levels in men and women. Appl. Physiol. Nutr. Metab..

[15] Roy M.-C., Anguenot R., Fillion C., Beaulieu M., Bérubé J., Richard D. (2011). Effect of a commercially-available algal phlorotannins extract on digestive enzymes and carbohydrate absorption in vivo. Food Res. Int..

[16] Baldrick F.R., McFadden K., Ibars M., Sung C., Moffatt T., Megarry K., Thomas K., Mitchell P., Wallace J.M.W., Pourshahidi L.K. (2018). Impact of a (poly)phenol-rich extract from the brown algae *Ascophyllum nodosum* on DNA damage and antioxidant activity in an overweight or obese population: A randomized controlled trial. Am. J. Clin. Nutr..

[17] Kim K.-T., Rioux L.-E., Turgeon S.L. (2014). α-amylase and α-glucosidase inhibition is differentially modulated by fucoidan obtained from *Fucus vesiculosus* and *Ascophyllum nodosum*. Phytochemistry.

[18] Kwon Y.-I.I., Vattem D.A., Shetty K. (2006). Evaluation of clonal herbs of Lamiaceae species for management of diabetes and hypertension. Asia Pac. J. Clin. Nutr..

[19] Maeda H., Hosokawa M., Sashima T., Takahashi N., Kawada T., Miyashita K. (2006). Fucoxanthin and its metabolite, fucoxanthinol, suppress adipocyte differentiation in 3T3-L1 cells. Int. J. Mol. Med..

[20] Zhang J., Tiller C., Shen J., Wang C., Girouard G.S., Dennis D., Barrow C.J., Miao M., Ewart H.S. (2007). Antidiabetic properties of polysaccharide- and polyphenolic-enriched fractions from the brown seaweed *Ascophyllum nodosum*. Can. J. Physiol. Pharmacol..

[21] De Martin S., Gabbia D., Carrara M., Ferri N. (2018). The brown algae *Fucus vesiculosus* and *Ascophyllum nodosum* reduce metabolic syndrome risk factors: A clinical study. Nat. Prod. Commun..

[22] Okimura T., Jiang Z., Liang Y., Yamaguchi K., Oda T. (2019). Suppressive effect of ascophyllan HS on postprandial blood sugar level through the inhibition of α-glucosidase and stimulation of glucagon-like peptide-1 (GLP-1) secretion. Int. J. Biol. Macromol..

[23] Chang Y., Ryu S., Zhang Y., Son H.J., Kim J.-Y., Cho J., Guallar E. (2012). A cohort study of serum bilirubin levels and incident non-alcoholic fatty liver disease in middle aged Korean workers. PLoS ONE.

[24] Luo L., An P., Jia X., Yue X., Zheng S., Liu S., Chen Y., An W., Winkler C.A., Duan Z. (2018). Genetically regulated bilirubin and risk of non-alcoholic fatty liver disease: A mendelian randomization study. Front. Genet..

[25] Viktorova J., Stranska-Zachariasova M., Fenclova M., Vitek L., Hajslova J., Kren V., Ruml T. (2019). Complex evaluation of antioxidant capacity of milk thistle dietary supplements. Antioxidants.

[26] Saha P., Talukdar A.D., Nath R., Sarker S.D., Nahar L., Sahu J., Choudhury M.D. (2019). Role of natural phenolics in hepatoprotection: A mechanistic review and analysis of regulatory network of associated genes. Front. Pharmacol..

[27] Šuk J., Jašprová J., Biedermann D., Petrásková L., Valentová K., Křen V., Muchová L., Vítek L. (2019). Isolated silymarin flavonoids increase systemic and hepatic bilirubin concentrations and lower lipoperoxidation in mice. Oxid. Med. Cell Longev..

[28] Stec D.E., John K., Trabbic C.J., Luniwal A., Hankins M.W., Baum J., Hinds T.D. (2016). Bilirubin binding to PPARα inhibits lipid accumulation. PLoS ONE.

[29] Gordon D.M., Blomquist T.M., Miruzzi S.A., McCullumsmith R., Stec D.E., Hinds T.D. (2019). RNA sequencing in human HepG2 hepatocytes reveals PPAR-α mediates transcriptome responsiveness of bilirubin. Physiol. Genom..

[30] Alkhouri N., Lawitz E., Noureddin M. (2019). Looking into the crystal ball: Predicting the future challenges of fibrotic NASH treatment. Hepatol. Commun..

[31] De Martin S., Gabbia D., Albertin G., Sfriso M.M., Mescoli C., Albertoni L., Paliuri G., Bova S., Palatini P. (2014). Differential effect of liver cirrhosis on the pregnane X receptor-mediated induction of CYP3A1 and 3A2 in the rat. Drug Metab. Dispos..

[32] Floreani M., Gabbia D., Barbierato M., De Martin S., Palatini P. (2012). Differential inducing effect of benzo[a]pyrene on gene expression and enzyme activity of cytochromes P450 1A1 and 1A2 in Sprague-Dawley and Wistar rats. Drug Metab. Pharmacokinet..

[33] Gabbia D., Pozzo L., Zigiotto G., Roverso M., Sacchi D., Dalla Pozza A., Carrara M., Bogialli S., Floreani A., Guido M. (2018). Dexamethasone counteracts hepatic inflammation and oxidative stress in cholestatic rats via CAR activation. PLoS ONE.

[34] Gabbia D., Pozza A.D., Albertoni L., Lazzari R., Zigiotto G., Carrara M., Baldo V., Baldovin T., Floreani A., Martin S.D. (2017). Pregnane X receptor and constitutive androstane receptor modulate differently CYP3A-mediated metabolism in early- and late-stage cholestasis. World J. Gastroenterol..

[35] Gabbia D., Roverso M., Guido M., Sacchi D., Scaffidi M., Carrara M., Orso G., Russo F.P., Floreani A., Bogialli S. (2019). Western diet-induced metabolic alterations affect circulating markers of liver function before the development of steatosis. Nutrients.

[36] Catanzaro D., Gabbia D., Cocetta V., Biagi M., Ragazzi E., Montopoli M., Carrara M. (2018). Silybin counteracts doxorubicin resistance by inhibiting GLUT1 expression. Fitoterapia.

[37] Castellani G., Paliuri G., Orso G., Paccagnella N., D’Amore C., Facci L., Cima F., Caicci F., Palatini P., Bova S. (2016). An intracellular adrenomedullin system reduces IL-6 release via a NF-κB-mediated, cAMP-independent transcriptional mechanism in rat thymic epithelial cells. Cytokine.

